# Veterinary Medical Association of New York County

**Published:** 1898-03

**Authors:** Robert W. Ellis

**Affiliations:** Secretary


### VETERINARY MEDICAL ASSOCIATION OF NEW YORK COUNTY.

THE regular monthly meeting was called to order at 8.30 o'clock, Wednesday evening, February 2d, at the Academy of Medicine, with the President, Dr. Huidekoper, presiding. The following members responded to roll-call: Drs. Bell, C. C. Cattanach, J. S. Cattanach, J. S. Cattanach, Jr., Dickson, Delaney, Ellis, Farley, Gill, Huidekoper, Hanson, Neher, O'Shea, Ryder, and Sherwood (15). There were also present as visitors Dr. Grenside, of New York; Drs. Ackerman and Goubeaud, of Brooklyn; Dr. Nicolas, of Mt. Vernon; and Messrs. English, Fretz, Fox, and Sandford, students from A. V. C.

Moved and seconded that guests be given the privilege of the floor in debate; carried.

Report of Judiciary Committee, Dr. O'Shea, chairman: Jury-exemption Bill was introduced in the Senate by Mr. Sullivan, January 5th, and referred to Codes Committee, the same having been introduced in the Assembly by Mr. Sullivan, January 5th, and referred to Codes Committee. The Judiciary Committee called the Civil Service Commission's attention to the law regarding the practice of veterinary medicine in New York State and appointments made, and they promise, if presented in writing, to see that the "commission" follow the spirit of the law regarding examinations for meat- and milk-inspectors.

The Judiciary is looking for illegal practitioners, and just as soon as sufficient evidence can be obtained they will be prosecuted. Moved and seconded that the report be accepted; carried.

Report of Committee on Ways and Means, Dr. Bell, chairman: This committee report that they are making arrangements for the five remaining meetings of the year, and have arrangements for the March meeting about complete. Moved and seconded that the report be accepted; carried.

Reading of papers: Dr. Neher read a carefully prepared paper on "Development of Bone," showing under the microscope the ossification zones or centres. Following this, Dr. Bell read a very interesting and practical paper on "Rubber Shoes for City Horses," which was freely discussed by the members present. During the reading of Dr. Bell's paper Dr. Neher was setting up his microscope, to which the members had free access during the rest of the evening.

Applications for membership in the Association were filed by Drs. Ackerman and Grenside; referred to the Board of Censors. Moved and seconded that a recess of twenty minutes be taken while the Board acted upon the applications; carried. Board of Censors favorably recommended Drs. Ackerman and Grenside, and they were declared members of the Association.

New Business: Moved and seconded that the Judiciary Committee formulate a letter, to be signed by the President and Secretary of the Association, to be sent to the President of the Civil Service Commission; carried.

Moved and seconded that a vote of thanks be extended to Drs. Neher nd Bell for their papers; carried.

Moved and seconded that the meeting adjourn; carried.

ROBERT W. ELLIS, D.V.S.,
Secretary.

